# Inhibition of the ATR-CHK1 signaling pathway sensitizes MCPyV-positive Merkel cell carcinoma to gemcitabine

**DOI:** 10.1186/s12885-026-16505-1

**Published:** 2026-07-16

**Authors:** Evelyn V. Proctor, Sherine F. Elsawa, Sarah R. Walker, Jingwei Cheng

**Affiliations:** Department of Molecular, Cellular, and Biomedical Sciences, University of New Hampshire, 46 College Rd, Rudman 206, Durham, NH 03824 USA

**Keywords:** ATR, CHK1, Gemcitabine, DNA damage response, Merkel cell carcinoma

## Abstract

Merkel cell carcinoma (MCC) is the primary cutaneous neuroendocrine carcinoma with a mortality rate higher than that of melanoma and other common skin cancers. In the United States, most cases of MCC are positive for Merkel cell polyomavirus (MCPyV), in which the viral small T antigen (ST) recruits the Tip60-EP400 chromatin remodeling complex to L-Myc, a MYC family protein with oncogenic properties. This complex initiates MYC-dependent transcriptional programs that induce replication stress. Given that ATR-CHK1 is crucial for responding to replication stress and maintaining genomic integrity, we investigated whether this ST-driven L-Myc/Tip60-EP400 program is associated with heightened reliance on ATR-CHK1 signaling in MCPyV^+^ MCC. Here, we demonstrate that MCPyV^+^ MCC cells exhibit a pronounced dependency on the ATR-CHK1 pathway and that combining CHK1 inhibition with the replication-blocking chemotherapeutic agent gemcitabine produces synergistic antiproliferative effects. In MCPyV^+^ MCC cell lines, the gemcitabine-CHK1 inhibitor combination markedly reduced cell viability and increased DNA damage and apoptosis compared to either agent alone. Conversely, in MCPyV^−^ cell lines, combination responses and apoptotic induction were attenuated, although some sensitivity to CHK1 inhibition alone was observed. These findings support ATR-CHK1 signaling as a candidate therapeutic vulnerability in MCPyV^+^ MCC and provide a rationale for further preclinical and translational evaluation of ATR or CHK1 inhibitors in combination with gemcitabine in this virally driven cancer.

## Significance statement

Virus-positive Merkel cell carcinoma (MCC) relies on ATR-CHK1 signaling to effectively manage replication stress. Disruption of this pathway enhances the susceptibility of tumor cells to gemcitabine, leading to increased DNA damage and apoptosis, whereas virus-negative cell lines exhibit a reduced response. These results indicate that combining ATR-CHK1 inhibition with gemcitabine warrants further evaluation in virus-positive MCC and suggest that MCPyV status may help stratify response.

## Introduction

Merkel cell carcinoma (MCC) is a rare but highly aggressive neuroendocrine skin cancer with a disease-specific mortality rate of approximately 33–46%, among the highest rates for skin cancers [1]. Clinically, MCC typically presents as a painless red or violaceous nodule on sun-exposed skin, with major risk factors including fair skin, advanced age, and immunosuppression [2–5]. The poor prognosis is partly attributable to late diagnosis, with approximately 30% of patients presenting with regional metastases [1].

Current treatments for MCC include surgery, radiation, chemotherapy, and immune checkpoint inhibitors [4]. Surgery and radiotherapy are effective for localized disease, although MCC carries a high risk of distant metastasis [4, 6]. Advanced or metastatic MCC responds poorly to conventional chemotherapy, with median progression-free and overall survival of approximately 3 and 9.5 months, respectively [7]. In contrast, immune checkpoint inhibitors targeting PD-1 or PD-L1 have substantially improved outcomes in MCC. In a phase II trial, the anti-PD-1 antibody pembrolizumab produced a 67% progression-free survival rate at 6 months among 26 patients with treatment-naive advanced MCC [8]. More broadly, PD-1/PD-L1 inhibitors have achieved an overall survival rate of approximately 65% in MCC, with durable responses observed in about half of patients with advanced disease [9]. However, a substantial proportion of patients either fail to respond or eventually relapse after checkpoint blockade [4, 8]. These limitations, together with the marked radiosensitivity and responsiveness of MCC to DNA-damaging therapies, highlight the need for strategies that combine targeted agents with DNA-damaging treatments to further improve outcomes in patients with advanced disease.

MCC can be categorized into two distinct subtypes: a virus-positive type associated with the Merkel cell polyomavirus (MCPyV) and a virus-negative type often linked to UV-induced DNA damage. In the United States, approximately 80% of MCC cases are MCPyV-positive (MCPyV^+^), whereas the remainder are MCPyV-negative (MCPyV^−^) [3, 4, 10]. MCPyV is a prevalent human polyomavirus, with 60–80% of the general population testing seropositive by early childhood; the virus typically resides asymptomatically in the skin [11, 12]. Although MCPyV infection is common and usually asymptomatic, progression to MCC is rare and requires clonal integration of the viral genome into the host chromosome [13]. Integration can occur through non-homologous end joining or microhomology-mediated break-induced replication, producing distinct integration patterns [14]. Immunosuppression is a well-established risk factor for MCC [15], consistent with a role for immune surveillance in controlling cells that express viral oncoproteins [16]. In MCPyV^+^ MCC tumors, the MCPyV genome is usually integrated and expresses a small T antigen (ST) and a truncated large T antigen (LT), whereas very few other recurrent driver mutations are found [17, 18]. ST is believed to drive cellular transformation [19, 20], whereas truncated LT supports tumor maintenance [21]. This pattern indicates that viral oncogene activity, rather than accumulated host mutations, is the primary driver of MCPyV^+^ MCC [4].

Recent investigations into primary tumors have found that MCPyV^+^ MCCs show increased ATR and CHK1 mRNA levels compared to MCPyV^−^ cases, consistent with upregulated DNA damage response signaling in virus-positive disease [22]. The ATR-CHK1 pathway is a crucial mediator of replication stress response. ATR (ataxia telangiectasia and Rad3-related kinase) and its downstream effector CHK1 function as safeguards during the S-phase by controlling DNA replication origin firing, stabilizing stalled replication forks, enforcing the S and G2/M checkpoints, and promoting DNA repair through homologous recombination. ATR is activated by replication stress, such as the accumulation of single-stranded DNA, and subsequently phosphorylates CHK1 (Ser345) to initiate checkpoint signaling [23–26]. This signaling can decelerate DNA synthesis, prevent new origin firing, and delay cell cycle progression, allowing cells time to resolve replication issues or, if damage is irreparable, to trigger apoptosis [26, 27]. ATR-CHK1 signaling also maintains genomic stability by facilitating DNA repair [28]. MCPyV^+^ MCC cells, burdened by viral-induced replication stress, likely depend on ATR-CHK1 activity for survival.

Inhibitors targeting the DNA damage response (DDR) have become increasingly prominent in cancer therapy [29]. Therapeutically, DDR inhibitors combined with other treatments can exploit synthetic lethality, wherein two sublethal insults together result in cell death [30]. Inhibitors of ATR, CHK1, and WEE1 (a G2 checkpoint kinase downstream of ATR/CHK1) have demonstrated single-agent antitumor activity in certain contexts. Importantly, preclinical studies have revealed that ATR, CHK1, and WEE1 inhibitors can work synergistically with immune checkpoint blockade or genotoxic chemotherapy in several cancers [23, 31]. Gemcitabine, a nucleoside analog that induces replication fork stalling and DNA damage [32], is a particularly attractive partner for ATR-CHK1 pathway inhibition. In several cancer types, gemcitabine combined with ATR or CHK1 inhibitors has shown promise in preclinical and early clinical studies, where ATR-CHK1 blockade enhances gemcitabine cytotoxicity by increasing replication-associated DNA damage and preventing checkpoint recovery [23, 33–36].

The mechanistic basis for the elevated replication stress in MCPyV^+^ MCC likely involves viral oncoprotein-driven transcriptional pathways. Array-based comparative genomic hybridization studies have demonstrated that L-Myc is recurrently amplified in MCC tumors [37]. Notably, MCPyV ST collaborates with the host oncogene L-Myc to recruit the Tip60-EP400 complex to E-boxes near the gene promoters [19]. The Tip60-EP400 complex integrates histone acetyltransferase and chromatin-remodeling functions to control chromatin organization and proliferation-associated gene expression [38–41]. ST, by recruiting the Tip60-EP400 complex to L-Myc, significantly elevates the transcription of a subset of MYC downstream targets [19, 38]. This heightened transcription can lead to transcription-replication conflicts and R-loop-associated fork stalling, resulting in replication stress [26]. Given that MYC overexpression is a well-established driver of oncogene-induced replication stress and dependence on ATR-CHK1 signaling [42, 43], the ST-driven L-Myc/Tip60-EP400 transcriptional axis is likely to contribute to the ATR-CHK1 dependency observed in MCPyV^+^ MCC.

In this study, we investigated the ATR-CHK1 pathway in MCPyV^+^ MCC using the ATR inhibitor ceralasertib, the CHK1 inhibitor prexasertib, and the WEE1 inhibitor adavosertib, hereafter referred to as ATRi, CHK1i, and WEE1i, respectively, in combination with gemcitabine. We hypothesized that MCPyV^+^ MCC cells, due to ST/L-Myc-induced replication stress, are more dependent on ATR-CHK1 signaling for survival and that inhibiting this pathway would sensitize the cells to replication stress-inducing chemotherapy. We further investigated whether the Tip60-EP400 complex (recruited by MCPyV ST/L-Myc) contributes to the replication stress phenotype.

## Materials and methods

### Cell lines and culture

The Merkel cell carcinoma (MCC) cell lines utilized in this study comprised MCPyV^+^ lines MKL-1, WaGa, and PeTa, as well as MCPyV^−^ lines UISO and MCC26. MKL-1 cells were generously provided by Masa Shuda (University of Pittsburgh). WaGa and UISO cells were obtained from Jürgen C. Becker (Medical University of Graz), PeTa was supplied by Roland Houben (University of Würzburg), and MCC26 was provided by J. Helen Leonard (University of Western Australia). An MKL-1 derivative with doxycycline-inducible short hairpin RNA (shRNA) against EP400 (MKL1-tet-pLKO-shEP400) was generated as described previously [44]. All cell lines were cultured in RPMI-1640 medium supplemented with 10% fetal bovine serum, 1% GlutaMAX, and 1% penicillin/streptomycin. Cultures were maintained at 37 °C in a 5% CO2 atmosphere and routinely tested for mycoplasma contamination using PCR, with negative results confirmed. Cells were passaged every 3–4 days at a density of 5 × 10^5^ cells/mL. MCPyV^+^ cell line identities were confirmed by Western blot detection of the viral LT antigen, which migrates at distinct molecular weights in each line due to cell line-specific truncation sites.

### Plasmid constructs

A short hairpin RNA (shRNA) targeting EP400 was cloned into the Tet-pLKO-puro inducible vector (Addgene, #21,915). The shEP400 insert sequence was 5′-GCAAAGACATCCACATATACA ctcgag TGTATATGTG GATGTCTTTG C TTTTT-3′, with lowercase letters indicating the loop. The shRNA cassette was inserted between the AgeI and EcoRI restriction sites of the vector. The resulting plasmid (Tet-pLKO-shEP400) was used to establish the MKL1-tet-pLKO-shEP400 cell line through lentiviral transduction and puromycin selection, as previously described [44].

### Reagents and inhibitors

Ceralasertib (ATRi), prexasertib (CHK1i), adavosertib (WEE1i), gemcitabine (Gem), and hydroxyurea (HU) were procured from Selleck Chemicals. Stock solutions were prepared in DMSO and stored at − 20 °C. Unless otherwise specified, working concentrations were as follows: ATRi 1 µM, CHK1i 10 nM, WEE1i 100 nM, Gem 10 nM, HU 500 µM. These concentrations were selected based on preliminary dose–response experiments (see the Results section). A summary of the inhibitors and their sources is provided in Table 1.

**Table 1:** Inhibitors used in this study and their sources

Inhibitor	Abbreviation	Vendor	Catalog Number
Adavosertib	WEE1i	Selleck Chemicals	S1525
Ceralasertib	ATRi	Selleck Chemicals	S7693
Gemcitabine	Gem	Selleck Chemicals	S1714
Hydroxyurea	HU	Selleck Chemicals	S1896
Prexasertib	CHK1i	Selleck Chemicals	S7178

### Cell viability assay

The assessment of cell proliferation and viability was conducted utilizing the CellTiter-Glo luminescent ATP assay (Promega). Prior to seeding, cells were treated with Accumax (Innovative Cell Technologies) to dissociate aggregates and subsequently plated in 96-well plates at a density of 2,000 cells per well. Inhibitors were administered either individually or in combination at specified concentrations. For single-agent dose–response experiments, cells were cultured for five days (ATRi, CHK1i, WEE1i) or four days (gemcitabine) in MCPyV^+^ cells. In MCPyV^−^ cells (UISO and MCC26), single-agent dose–response experiments were performed over five days for all inhibitors including gemcitabine. Combination index experiments were performed over four days. Each experimental condition was evaluated in six technical replicates across a minimum of three independent biological experiments. Luminescence was quantified using a SpectraMax plate reader (Molecular Devices) and normalized against the vehicle control wells. For combination treatments, the CompuSyn software was employed to calculate the Combination Index (CI) values and generate isobolograms [45]. In this analytical framework, a CI value of 1 denotes an additive drug interaction, a CI value of less than 1 indicates synergy (with CI values below 0.7 representing strong synergy), and a CI value of greater than 1 signifies antagonism.

### Western blotting

Cells were exposed to the specified pharmacological agents for 24 h, unless otherwise indicated, and then lysed in RIPA buffer supplemented with protease and phosphatase inhibitors. Protein concentrations were quantified using the BCA assay (Thermo Fisher Scientific). Equal protein amounts (30–60 µg per sample) were separated by SDS-PAGE on 4–20% gradient gels (Bio-Rad) and transferred onto PVDF membranes. Membranes were blocked with 5% non-fat dry milk in 1 × TBST (150 mM NaCl, 0.1% (v/v) Tween 20, 10 mM Tris, pH 7.5) for 1 h at room temperature, then incubated with primary antibodies diluted in 3% BSA in 1 × TBST overnight at 4 °C. After being washed 5 times with 1 × TBST, membranes were incubated with HRP-conjugated secondary antibodies for 1 h at room temperature and washed 5 additional times with 1 × TBST. Signals were detected using an ECL substrate (Bio-Rad) and visualized using a Bio-Rad ChemiDoc system. Band intensities were quantified using the ImageJ software. Primary antibodies against ATR, phospho-ATR Thr1989, CHK1, phospho-CHK1 S345, CDK1, phospho-CDK1 Y15, RPA32, phospho-RPA32 S33, KAP1, phospho-KAP1 S824, γH2AX (phospho-Ser139 H2AX), PARP, EP400, and GAPDH were obtained from Cell Signaling Technology or Bethyl Laboratories (refer to Table 2 for antibody information and dilutions). Densitometry values were normalized to GAPDH or the corresponding total protein, as appropriate. Data represent at least 3 independent biological replicates.

**Table 2:** Primary and secondary antibodies used for Western blotting, along with host species, dilution, vendor, and catalog number

	Host Species	Dilution	Vendor	Catalog Number
Antibody (Primary)				
ATR	Rabbit	1:5000	Cell Signaling	2790S
CDK1	Rabbit	1:5000	Cell Signaling	77055S
CHK1	Mouse	1:5000	Cell Signaling	2360S
EP400	Rabbit	1:1000	Bethyl	A300-541A
GAPDH	Rabbit	1:5000	Cell Signaling	5174S
KAP1	Rabbit	1:5000	Cell Signaling	4123S
PARP	Rabbit	1:5000	Cell Signaling	9542S
p-ATR (Thr1989)	Rabbit	1:1000	Cell Signaling	30632S
p-CDK1	Rabbit	1:5000	Cell Signaling	4539S
p-CHK1	Rabbit	1:5000	Cell Signaling	2348S
p-KAP1	Rabbit	1:1000	Cell Signaling	4127S
p-RPA32	Rabbit	1:1000	Cell Signaling	10148S
RPA32	Rabbit	1:5000	Cell Signaling	35869S
Vinculin	Mouse	1:30,000	Sigma Aldrich	V9131
γH2AX	Mouse	1:1000	Cell Signaling	80312S
γH2AX	Rabbit	1:1000	Cell Signaling	2577S
Caspase 3	Rabbit	1:5000	Cell Signaling	9662S
Cleaved Caspase 3	Rabbit	1:1000	Cell Signaling	9664S
Antibody (Secondary)				
Anti-rabbit HRP-conjugated		1:5000	Cell Signaling	7074S
Anti-mouse HRP-conjugated		1:5000	Cell Signaling	7076S

### Apoptosis assay (flow cytometry)

MCC cells were treated with the indicated drugs for 24 h, followed by assessment of apoptosis using Annexin V-FITC and propidium iodide (PI) staining. Apoptosis was measured using the FITC Annexin V Apoptosis Detection Kit II (BD Pharmingen, 556,570). Approximately 1.5 × 10^6^ cells per condition were washed with PBS and resuspended in 1 × binding buffer. FITC-conjugated Annexin V and PI were added to each sample according to the manufacturer’s instructions and incubated for 15 min at room temperature in the dark. Prior to acquisition, the samples were passed through a 40 µm nylon mesh to remove cell clumps. The stained cells were analyzed using a Sony SH800Z flow cytometer. Compensation and quadrant gating were established using unstained and single-stained controls. For each treatment, 1 × 10^5^ events were collected and analyzed using FlowJo software (version 10.10). In the dot plots, Annexin V^+^/PI^−^ cells were classified as early apoptotic, Annexin V +/PI + as late apoptotic, Annexin V^−^/PI^+^ as necrotic or damaged, and double-negative cells as viable. Data represent at least three independent biological replicates. The percentages of apoptotic (early and late) and viable cells were quantified and compared across the treatments.

### Cell cycle analysis (flow cytometry)

Cell-cycle distribution was evaluated by PI staining of DNA content, following a protocol adapted from Matissek et al. [46]. Briefly, treated cells (1.5 × 10^6^) were collected, washed with ice-cold PBS, and resuspended in 1 mL ice-cold PBS. While vortexing, 9 mL of ice-cold fixation buffer (70% ethanol, 30% PBS) was added dropwise. After fixation for at least 2 h at − 20 °C, cells were washed with ice-cold PBS and resuspended in PI staining solution (0.1% Triton X-100, 100 µg/mL RNase A, and 20 µg/mL PI) for 30 min at 37 °C. Flow cytometry data were acquired within 1 h of staining using a Sony SH800Z flow cytometer and analyzed using FlowJo software (version 10.10). For each treatment, 1 × 10^5^ events were collected. Doublets were excluded by FSC-A versus FSC-H gating before cell cycle analysis. The cell populations corresponding to the sub-G1 (apoptotic), G0/G1, S, and G2/M phases were quantified. All cell cycle experiments were conducted in biological triplicates.

For all flow cytometry experiments, debris was excluded by gating on forward scatter versus side scatter (FSC vs. SSC), and doublets were excluded by gating on single cells using forward scatter area versus height (FSC-A vs. FSC-H) before analysis.

### Statistical analysis

Data are expressed as mean ± standard deviation (SD). Statistical significance was assessed using GraphPad Prism Version 10.6.0. One-way ANOVA followed by Tukey’s multiple-comparisons test was used to determine statistical significance for densitometry and flow cytometry data. Significance levels: *p < 0.05, **p < 0.01, ***p < 0.001, and ****p < 0.0001.

## Results

### Gemcitabine and CHK1 inhibition synergistically reduce MCPyV^+^ MCC cell viability

To identify effective combination strategies targeting the ATR-CHK1 pathway in MCC, we first established baseline sensitivity of the MCPyV^+^ cell line MKL-1 to single-agent ceralasertib (ATRi), prexasertib (CHK1i), and adavosertib (WEE1i). Working concentrations of approximately 1 µM for ceralasertib, 10 nM for prexasertib, and 100 nM for adavosertib were selected from preliminary dose–response experiments. These concentrations were used in subsequent combination experiments.

**Fig. 1:**
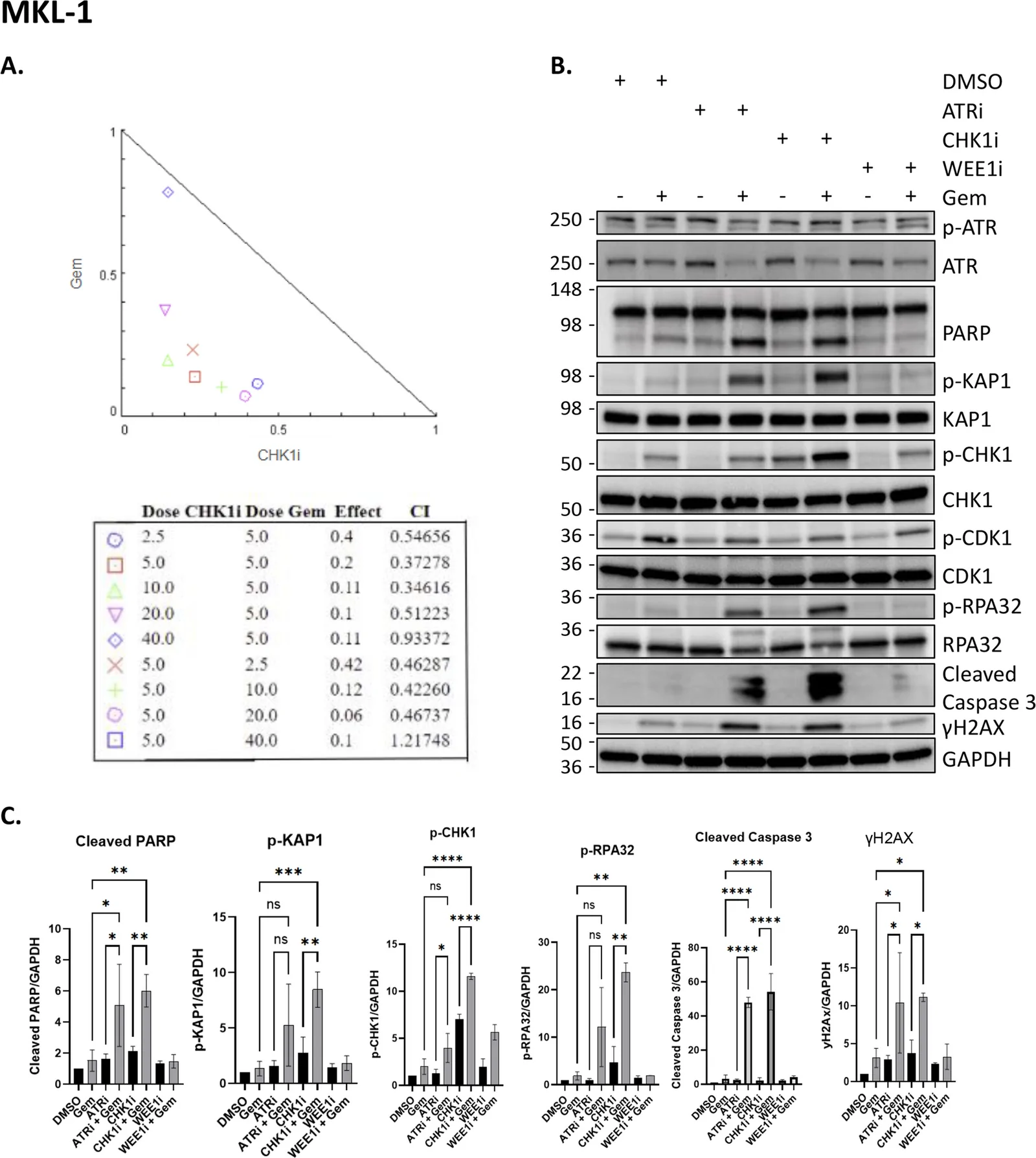
Gemcitabine combined with ATR-CHK1 pathway inhibitors produces synergistic effects in MCPyV^+^ MCC cells. **A** Isobologram of MKL-1 cells treated with gemcitabine (Gem) and prexasertib (CHK1i) for 4 days. Data points below the line of additivity (diagonal line) indicate synergy. Combination index (CI) values less than 1, shown for each dose pair, confirm synergistic interactions at most concentrations tested. **B** Western blot analysis of DNA damage and apoptosis markers in MKL-1 cells treated for 24 h with vehicle (DMSO), Gem (10 nM), ceralasertib (ATRi, 1 µM), prexasertib (CHK1i, 10 nM), adavosertib (WEE1i, 100 nM), or gemcitabine combined with ATRi, CHK1i, or WEE1i. Immunoblots show phosphorylated ATR (p-ATR Thr1989), ATR, p-CHK1 (Ser345), CHK1, p-RPA32 (Ser33), RPA32, p-KAP1 (Ser824), KAP1, γH2AX, p-CDK1 (Tyr15), CDK1, cleaved PARP, and cleaved caspase 3, with GAPDH as a loading control. Gem + CHK1i produced the strongest increases in p-CHK1, p-RPA32, p-KAP1, and γH2AX, along with substantial PARP cleavage and cleaved caspase 3; Gem + ATRi also increased p-KAP1, γH2AX, PARP cleavage, and cleaved caspase 3, with more modest changes in p-CHK1 and p-RPA32. Inhibitor concentrations correspond to 5-day IC50 values and are not expected to produce complete acute target suppression at this 24-h time point. **C** Quantification of cleaved PARP, p-KAP1, p-CHK1, p-RPA32, cleaved caspase 3, and γH2AX band intensity from blots as in panel B, normalized to GAPDH. Bars represent the mean ± SD (*n* = 3). Significance for densitometry data was determined by one-way ANOVA with post hoc Tukey test

To test whether combining these inhibitors with gemcitabine produces synergistic effects, we first determined the gemcitabine IC50 from viability assays, which was approximately 10 nM in MKL-1 cells (Supplementary Figure S1A). Cells were then treated with gemcitabine and CHK1i using the standard combination index (CI) methodology: one-half the IC50 of one drug was combined with 0.25 x, 0.5 x, 1 x, 2 x, or 4 × the IC50 of the other drug, and vice versa. CI analysis revealed that all three combinations exhibited synergism (CI < 1) across most dose pairs. Gemcitabine plus CHK1i showed seven dose pairs with strong synergy (CI < 0.7) (Fig. 1A), gemcitabine plus ATRi showed eight (Supplementary Figure S1B), and gemcitabine plus WEE1i showed five (Supplementary Figure S1C). At the highest gemcitabine concentration (40 nM), the CI approached 1, likely reflecting near-maximal cytotoxicity from gemcitabine alone. Parallel experiments in WaGa cells (MCPyV^+^) showed similar synergistic patterns for all three combinations (Supplementary Figures S2A, S3), indicating that this synergy is not cell line-specific.

To characterize the cellular consequences of combination treatment, we performed Western blot analysis on MKL-1 cells treated for 24 h with single agents or combinations at the IC50-based doses described above. Blots were probed for replication stress signaling markers (p‑ATR Thr1989, p‑CHK1 Ser345, p‑RPA32 Ser33), DNA damage markers (p‑KAP1 Ser824, γH2AX), cell cycle regulation (p-CDK1 Tyr15), and apoptosis markers (cleaved PARP, cleaved caspase 3). Single-agent gemcitabine or ATR-CHK1 pathway inhibitors produced only modest changes in these readouts at the concentrations used. In contrast, combination treatments increased DNA damage markers and apoptosis compared to either agent alone (Fig. 1B). Gem + CHK1i showed the most prominent p-CHK1 Ser345 and p-RPA32 Ser33 signals, together with robust increases in p-KAP1 Ser824, γH2AX, cleaved PARP, and cleaved caspase 3. Gem + ATRi also increased p-KAP1, γH2AX, cleaved PARP, and cleaved caspase 3, indicating enhanced DNA damage and apoptosis, while p-CHK1 and p-RPA32 showed more modest changes at this 24-h time point. The p-CHK1 and p-RPA32 signals in the Gem + ATRi condition are best interpreted as net readouts of ongoing replication stress and DNA damage under incomplete checkpoint control, rather than as direct pharmacodynamic measures of ATR catalytic activity, as ATM and DNA-PKcs can also phosphorylate CHK1 Ser345 and RPA32 Ser33 in the context of replication-associated double-strand breaks [47, 48]. Densitometric quantification confirmed that these markers were significantly elevated in the Gem + ATRi and/or Gem + CHK1i conditions relative to single-agent treatments (Fig. 1C), supporting the conclusion that checkpoint inhibition converts gemcitabine-induced replication stress into extensive DNA damage and apoptosis. We also observed increased inhibitory CDK1 phosphorylation (Tyr15) under gemcitabine-containing conditions, consistent with checkpoint engagement. Western blot analysis of WaGa cells yielded a similar pattern (Supplementary Figure S2B, C), indicating that the combination-induced replication stress, DNA damage signaling, and apoptosis are reproducible across MCPyV^+^ cell lines.

In contrast, gemcitabine plus WEE1i produced only modest increases in DNA damage markers and minimal PARP cleavage in MKL-1 and WaGa cells (Fig. 1B, Supplementary Figure S2B). Given this weaker pro-apoptotic activity compared with gemcitabine plus ATRi or CHK1i, we focused subsequent functional experiments on the ATRi and CHK1i combinations.

### ATR-CHK1 inhibition combined with gemcitabine enhances cytotoxicity and apoptosis in MCPyV^+^ MCC

Having established biochemical evidence of enhanced DNA damage and apoptosis with combination treatment, we next quantified these effects using flow cytometry. Because MKL-1 cells tend to aggregate, which impedes accurate flow cytometric analysis, we used WaGa and PeTa cells for apoptosis and cell cycle experiments.

**Fig. 2:**
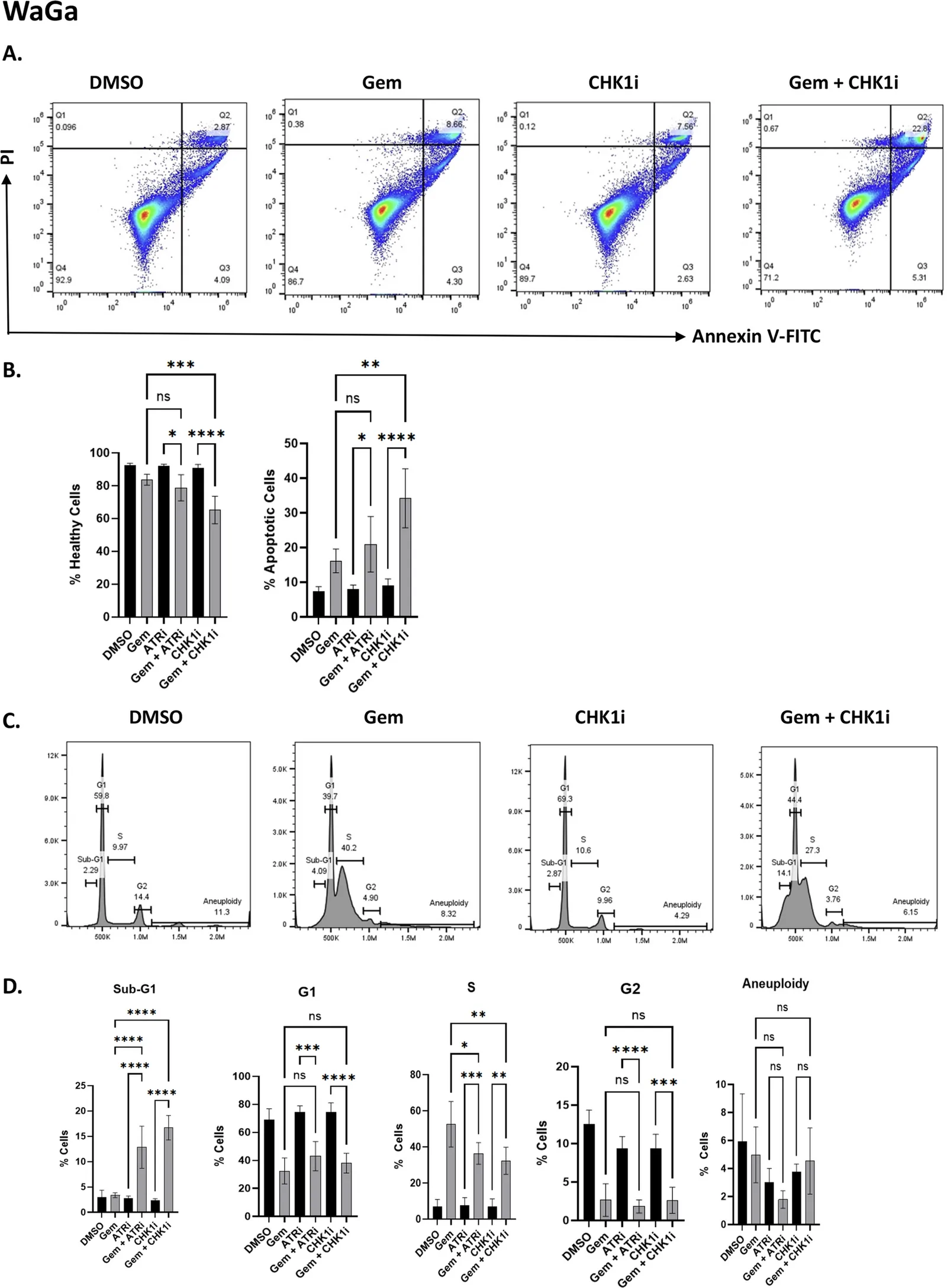
ATR-CHK1 inhibition combined with gemcitabine induces apoptosis and S-phase arrest in MCPyV^+^ MCC cells. **A** Representative Annexin V/PI flow cytometry dot plots for WaGa cells treated for 24 h with DMSO, Gem (10 nM), CHK1i (10 nM), or Gem + CHK1i. Quadrants: Q4 (Annexin V⁻/PI⁻), viable; Q3 (Annexin V^+^/PI⁻), early apoptotic; Q2 (Annexin V^+^/PI^+^), late apoptotic; Q1 (Annexin V⁻/PI^+^), necrotic. Gem + CHK1i produces the highest percentage of Annexin V^+^ (apoptotic) cells. Representative plots of the ATRi conditions are shown in Supplementary Figure S4A. **B** Quantification of apoptosis under all conditions, including ATRi and Gem + ATRi. Bars represent the mean percentage of cells ± SD (*n* = 4) in the viable or apoptotic (early + late) categories. Gem + ATRi and Gem + CHK1i significantly increase apoptotic fractions and decrease viable cells compared to single agents. **C** Representative cell cycle histograms (PI DNA content) of WaGa cells treated as described in (**A**). Gemcitabine induces S-phase accumulation; Gem + CHK1i maintains elevated S-phase while increasing sub-G₁ events, corresponding to apoptotic DNA fragmentation. Representative histograms of the ATRi conditions are shown in Supplementary Figure S4B. **D** Quantification of cell cycle phase distribution under all conditions (mean ± SD, *n* = 4). Gemcitabine and combination treatments elevated the S-phase. Gem + ATRi and Gem + CHK1i significantly increase the sub-G1 (apoptotic) population. Significance was determined by one-way ANOVA followed by Tukey's multiple-comparisons test

Annexin V/PI staining revealed that single-agent gemcitabine induced modest apoptosis, increasing the Annexin V^+^ fraction from approximately 7% in DMSO controls to about 13% at 24 h, whereas single-agent ATRi or CHK1i had minimal effects (Fig. 2A, Supplementary Figure S4A). In contrast, combination treatments markedly increased apoptosis: Gem + CHK1i increased Annexin V^+^ cells to approximately 28%, representing approximately a two-fold increase over gemcitabine alone (*p* < 0.0001; Fig. 2B, Supplementary Figure S4B). Gem + ATRi also significantly enhanced apoptosis, albeit to a slightly lesser extent. Correspondingly, the percentage of viable cells was significantly reduced in the Gem + CHK1i group compared to that in the gemcitabine alone group (*p* < 0.001). Similarly, PeTa cells (MCPyV^+^) showed numerically higher Annexin V + fractions with combination treatments compared with gemcitabine alone, although these differences did not reach statistical significance because of high baseline apoptosis and variability (Supplementary Figure S5). Together with PARP cleavage data from MKL-1 and WaGa cells (Fig. 1B; Supplementary Figure S2B), these results demonstrate that ATR-CHK1 inhibition potentiates gemcitabine-induced apoptosis in multiple MCPyV^+^ cell lines.

Cell cycle analysis revealed that gemcitabine alone significantly increased the S-phase fraction in WaGa cells compared to the controls, consistent with replication fork stalling (Fig. 2C, D). Single-agent ATRi or CHK1i had minimal effects at this time point. Combination treatments maintained elevated S-phase populations while substantially increasing the sub-G1 (apoptotic) fraction, producing roughly twofold (Gem + ATRi) and threefold (Gem + CHK1i) increases relative to gemcitabine alone (*p* < 0.0001 for both combinations). G1-phase populations correspondingly decreased with all gemcitabine-containing treatments, and G2 percentages were slightly reduced, consistent with fewer cells completing the S phase. PeTa cells exhibited a similar pattern, with increased S-phase and sub-G1 fractions in combination-treated cells (Supplementary Figure S6). Of note, sub-G1 accumulation in PeTa cells was modest relative to their high baseline apoptotic fraction detected by Annexin V/PI staining (Supplementary Figure S5), which may reflect loss of fragile apoptotic cells during the ethanol fixation and washing steps required for cell cycle analysis. Neither cell line exhibited significant differences in aneuploidy between treatments. These data indicate that ATR-CHK1 inhibition enhances the cytotoxic consequences of gemcitabine-induced replication stress in MCPyV^+^ MCC cells, consistent with increased apoptotic output across multiple assays.

### MCPyV^−^ MCC cells show attenuated synergistic responses to gemcitabine plus ATR-CHK1 inhibition

The preceding experiments established that MCPyV^+^ MCC cells are highly sensitive to combined ATR-CHK1 inhibition and gemcitabine. We next asked whether this sensitivity is specific to virus-positive cells. To address this, we tested two MCPyV^−^ MCC lines, UISO and MCC26, which lack viral oncoproteins and instead harbor UV-signature mutations. Single-agent viability assays revealed that UISO sensitivity to ATRi (IC50 ~ 1 µM) and WEE1i (IC50 ~ 100 nM) was comparable to MCPyV^+^ MKL-1 cells, whereas UISO displayed slightly greater sensitivity to single-agent CHK1i, with an IC50 of ~ 5 nM compared with ~ 10 nM in MCPyV^+^ cells (Supplementary Figure S7A).

**Fig. 3:**
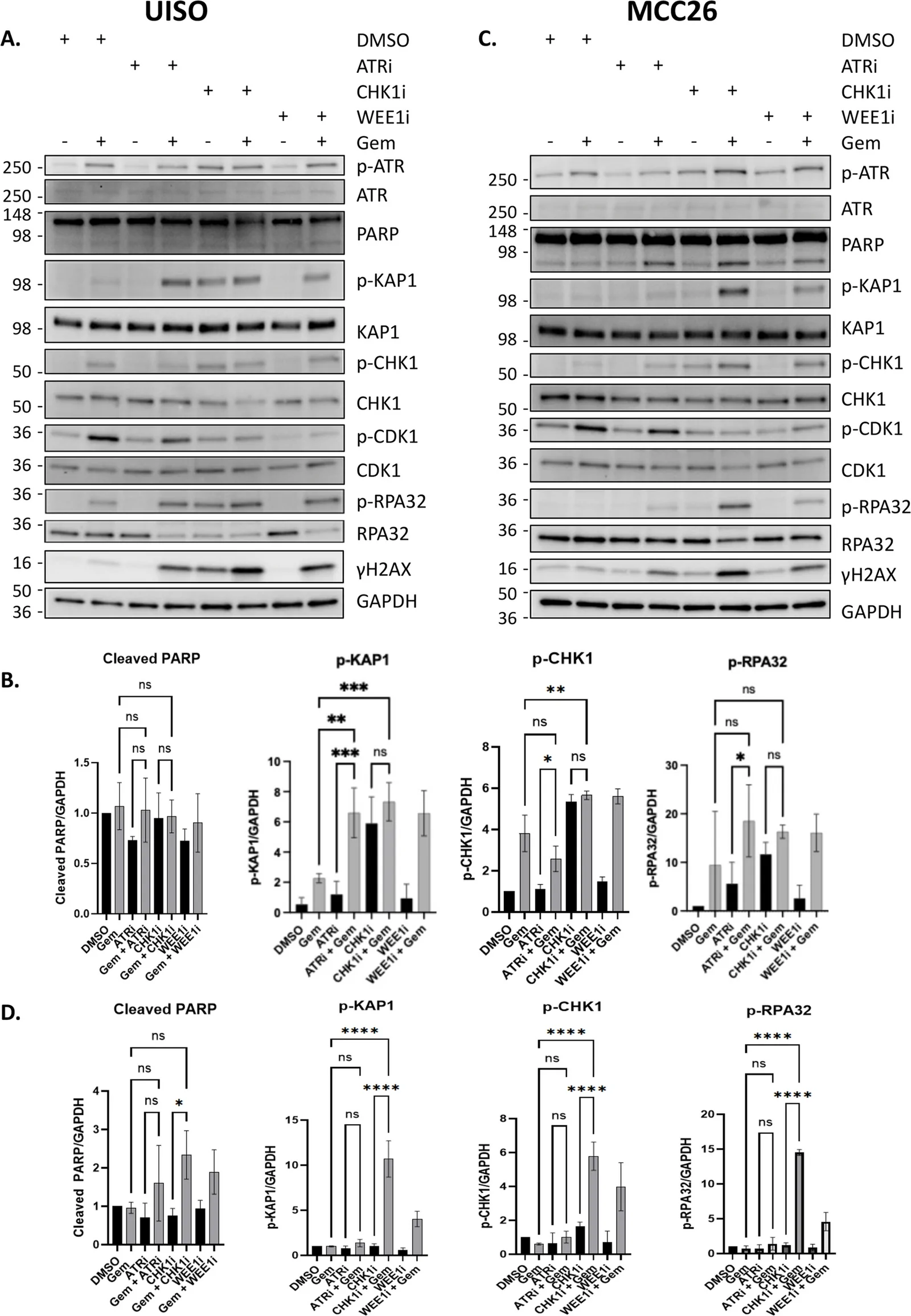
MCPyV^−^ MCC cells exhibit blunted responses to ATR-CHK1 inhibition in combination with gemcitabine. **A** Western blot analysis of UISO cells (MCPyV^−^) treated for 24 h with DMSO, Gem (10 nM), ATRi (1 µM), CHK1i (10 nM), WEE1i (100 nM), or the indicated combinations. Immunoblots were probed with the indicated antibodies. Unlike MCPyV^+^ cells (Fig. 1B), UISO cells showed limited gemcitabine-dependent amplification of checkpoint/replication-stress signaling: CHK1i induced p-CHK1, p-RPA32, and p-KAP1, but the addition of gemcitabine did not significantly further increase these markers relative to CHK1i alone, and PARP cleavage remained minimal. **B** Quantification of cleaved PARP, p-KAP1, p-CHK1, and p-RPA32 from UISO blots as in (**A**), normalized to GAPDH (mean ± SD, *n* = 3). **C** Western blot analysis of MCC26 cells (MCPyV^−^) treated as described in (A). At the fixed doses used, ATRi + Gem produced little change in p-CHK1, p-RPA32, and p-KAP1 compared to ATRi alone, whereas CHK1i + Gem robustly increased the phosphorylation of CHK1, RPA32, and KAP1. Despite this signaling, PARP cleavage was barely detectable. **D** Quantification of cleaved PARP, p-KAP1, p-CHK1, and p-RPA32 from MCC26 blots as in **C**, normalized to GAPDH (mean ± SD, *n* = 3). Significance for densitometry data was determined by one-way ANOVA with post hoc Tukey test

Despite this single-agent sensitivity, Western blot analysis and densitometry revealed an attenuated gemcitabine-dependent replication-stress escalation in UISO cells (Fig. 3A, B). In MCPyV^+^ cells, Gem + CHK1i strongly increased p-CHK1 compared to CHK1i alone, consistent with ATR hyperactivation under unresolved replication stress (Fig. 1B, C). In contrast, in UISO cells, CHK1 inhibition induced p-CHK1 (Ser345) along with increases in p-RPA32 and p-KAP1; however, the addition of gemcitabine to CHK1i did not significantly increase these readouts relative to CHK1i alone (bar 5 vs. bar 6). Gemcitabine alone (and WEE1i + gemcitabine) induced measurable p-CHK1 expression; however, cleaved PARP was not detected under any condition, indicating a limited apoptotic response. Modest γH2AX induction was observed with gemcitabine-containing treatments (Fig. 3A). CI analysis confirmed attenuated synergy in UISO cells: only three of nine dose pairs for Gem + ATRi and four of nine for Gem + CHK1i achieved strong synergy (CI < 0.7), compared with seven or more in the MCPyV^+^ lines, and several dose pairs exhibited antagonistic interactions (CI > 1) (Supplementary Figure S8A).

To confirm these findings, we tested MCC26, a second MCPyV^−^ cell line. MCC26 displayed greater inhibitor resistance: IC50 values were approximately 15-fold higher for ATRi, 16-fold higher for CHK1i, and fourfold higher for WEE1i compared to MCPyV^+^ cells (Supplementary Figure S7B). In MCC26, ATR inhibition at 1 µM produced minimal changes in p-KAP1, p-CHK1, and p-RPA32, and addition of gemcitabine did not significantly augment these markers (bar 3 vs bar 4; Fig. 3C, D), consistent with relative ATRi resistance under these fixed-dose conditions. In contrast, CHK1i + Gem (and to a lesser extent WEE1i + Gem) induced replication-stress/DNA damage signaling (p-CHK1, p-RPA32, and p-KAP1), yet PARP cleavage remained barely detectable (Fig. 3C). CI analysis of MCC26 cells revealed a more nuanced pattern. Gem + CHK1i showed only three of nine dose pairs with strong synergy (CI < 0.7), with the majority exhibiting antagonism (Supplementary Figure S8B). Gem + ATRi produced CI values consistently below 1 (range 0.09–0.66); however, the fraction of cells affected at all dose pairs was minimal (0.02–0.06), indicating that only 2–6% of cells were affected even at ATRi concentrations approximately tenfold higher than those producing robust responses in MCPyV^+^ cells. Thus, while a mathematical synergistic interaction is detectable, the absolute cytotoxic effect is negligible at these concentrations, consistent with the minimal pathway engagement and absent PARP cleavage observed at the 1 µM ATRi dose used for functional assays (Fig. 3C, D, Supplementary Figure S8B). This indicates that replication-stress signaling does not efficiently translate into apoptosis in these MCPyV^−^ models under the conditions tested.

Together, the two MCPyV^−^ models exhibited heterogeneous single-agent sensitivities and replication-stress responses, which may reflect the genetically diverse, UV-driven pathogenesis of virus-negative MCC. Despite this heterogeneity, the apoptotic response to combination treatment was less pronounced in both MCPyV^−^ lines than in the MCPyV^+^ models, as PARP cleavage remained limited under conditions that robustly induced apoptosis in virus-positive cells. These data suggest that MCPyV status may influence sensitivity to ATR-CHK1-targeted combination therapy, although additional factors likely contribute to the variable responses observed among MCPyV^−^ models.

### Tip60-EP400 contributes to ATR-CHK1 activation during replication stress

MCPyV small T antigen recruits the Tip60-EP400 chromatin-remodeling complex to L-Myc, driving transcriptional programs that may generate replication stress. To test whether Tip60-EP400 contributes to ATR-CHK1 dependency in MCPyV^+^ MCC, we used MKL-1 cells with doxycycline-inducible knockdown of E1A binding protein p400 (EP400), the ATPase subunit of the complex. Because this derivative line was generated by stable lentiviral integration, absolute signaling intensities are not directly comparable to those of the parental MKL-1 cells shown in Fig. 1; all comparisons in this section are made within the isogenic system. We previously showed that EP400 knockdown significantly reduces viability in MCPyV^+^ MKL-1 cells but has no effect on the MCPyV^−^ line UISO [19], consistent with a virus-specific requirement for this complex.

We first assessed whether EP400 depletion alters sensitivity to ATR-CHK1 pathway inhibitors. As expected, EP400 knockdown reduced cell viability, given its role in proliferation. To isolate the effect of EP400 on drug sensitivity, we normalized inhibitor-treated EP400-knockdown cells to EP400-knockdown alone. Under these conditions, the dose–response curves for ATRi, CHK1i, and WEE1i were nearly identical between control and EP400-knockdown cells (Supplementary Figure S9A), indicating that EP400 does not measurably change the sensitivity to these inhibitors beyond its direct impact on cell growth.

**Fig. 4:**
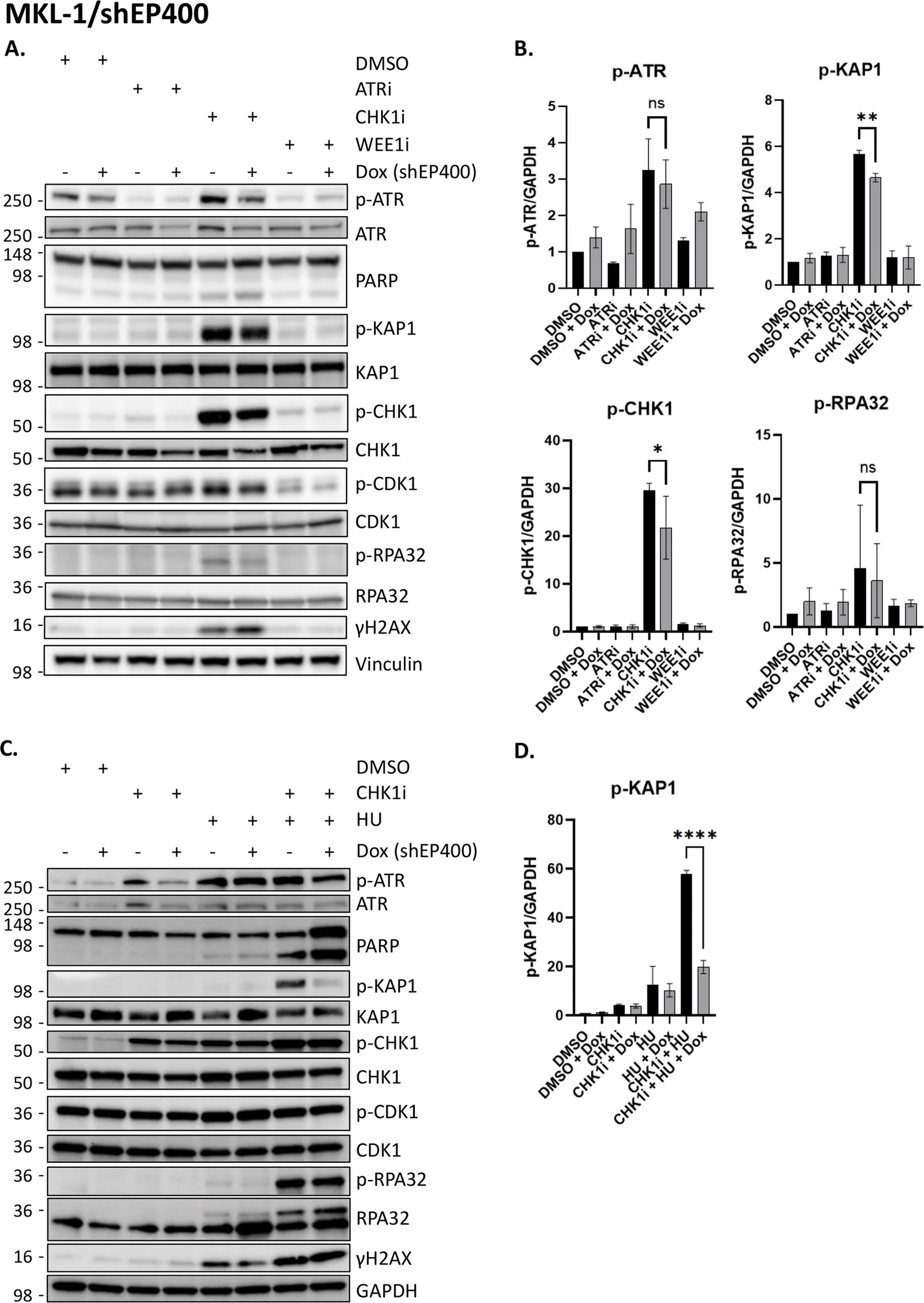
Tip60-EP400 contributes to ATR-CHK1 activation during replication stress in MCPyV^+^ MCC cells. **A** Western blot analysis of MKL-1 cells with doxycycline (Dox)-inducible EP400 knockdown. Cells were cultured ± Dox for 4 days, then treated with DMSO, ATRi (1 µM), CHK1i (10 nM), or WEE1i (100 nM) for 24 h. Immunoblots were probed with the indicated antibodies. In control cells (Dox-), CHK1i markedly induced p-ATR, p-CHK1, p-KAP1, p-RPA32, and γH2AX. In EP400-knockdown cells (Dox +), CHK1i still induced these markers, but the magnitude of induction was reduced. **B** Quantification of p-ATR, p-KAP1, p-CHK1, and p-RPA32 from experiments as in (A), normalized to GAPDH and expressed relative to the DMSO control (mean ± SD, *n* = 3). Black bars, control (Dox −); gray bars, EP400 knockdown (Dox +) cells. CHK1i-induced p-CHK1 (*p < 0.05) and p-KAP1 (**p < 0.01) levels were significantly reduced in EP400-knockdown cells. Changes in p-ATR and p-RPA32 levels were not statistically significant (ns). **C** Western blot analysis of MKL-1-shEP400 cells cultured ± Dox, then treated with CHK1i (10 nM) ± hydroxyurea (HU, 500 µM) for 24 h. Immunoblots were probed with the indicated antibodies. HU alone induced p-ATR, p-CHK1, and γH2AX; CHK1i + HU further increased p-RPA32 and γH2AX and induced PARP cleavage. In EP400-knockdown cells, CHK1i + HU produced a similar induction of most markers, but p-KAP1 was visibly reduced compared to that in control cells. **D** Quantification of p-KAP1 from experiments as in (C) (mean ± SD, *n* = 3). Significance for densitometry data was determined by one-way ANOVA with post hoc Tukey test

We next examined whether EP400 knockdown alters DNA damage signaling during CHK1 inhibition. In control MKL-1 cells, 24-h CHK1i treatment markedly increased p-ATR, p-CHK1, p-KAP1, p-RPA32, and γH2AX compared with DMSO, consistent with replication stress following CHK1 blockade. In EP400-knockdown cells, CHK1i still induced these markers, but the magnitude of induction was reduced; in particular, p-CHK1 and p-KAP1 induction were significantly decreased relative to control cells, while changes in p-ATR and p-RPA32 did not reach statistical significance (Fig. 4A, B). These findings suggest that Tip60-EP400 can modulate ATR-CHK1 pathway activation under conditions of oncogene-driven endogenous replication stress in MCPyV^+^ MCC.

To test whether EP400's contribution becomes more apparent under heightened stress, we combined CHK1i with hydroxyurea (HU), which induces replication fork stalling by depleting nucleotide pools and activates ATR-CHK1 signaling in MCC cells [49]. In control cells, CHK1i plus HU strongly increased p-KAP1, p-RPA32, γH2AX, and PARP cleavage levels. In EP400-knockdown cells, CHK1i plus HU still robustly induced p-RPA32, γH2AX, and PARP cleavage, but p-KAP1 induction was significantly reduced compared with control cells (Fig. 4C, D), indicating a selective attenuation of the p-KAP1 damage-signaling response, despite robust induction of other replication stress and apoptosis markers. Together, these results indicate that Tip60-EP400 contributes to the replication stress burden in MCPyV^+^ cells and modulates the magnitude of ATR-CHK1 pathway activation, but its loss does not independently alter drug sensitivity.

## Discussion

MCC is an aggressive skin cancer with limited treatment options beyond immune checkpoint inhibitors. Here, we demonstrate that MCPyV^+^ MCC cells display heightened sensitivity to combined ATR-CHK1 pathway inhibition and gemcitabine, providing a potential therapeutic strategy for virus-positive MCC. This vulnerability appears to stem from viral oncoprotein-driven replication stress, which creates a dependency on ATR-CHK1 that can be exploited through synthetic lethality.

### Mechanistic basis of the synergy

Our data support a model in which gemcitabine-induced replication fork stalling becomes cytotoxic when ATR-CHK1 checkpoint function is pharmacologically compromised. Normally, ATR-CHK1 signaling stabilizes stalled forks, limits replication-stress escalation, and permits recovery. When this protective response is blocked, cells accumulate replication-associated lesions that culminate in apoptosis. The observation that neither gemcitabine nor ATRi/CHK1i alone produced strong apoptotic signaling, whereas the combinations did, is consistent with a synthetic lethal interaction driven by checkpoint abrogation during drug-induced replication stress.

### Gemcitabine and ATR-CHK1 inhibitor combinations

Although gemcitabine is occasionally used for MCC, no prior studies have explored its combination with ATR or CHK1 inhibitors in MCC. This combination has shown promise in other malignancies: preclinical studies in pancreatic cancer demonstrated synergistic cell killing [33], clinical trials in ovarian cancer showed biomarker-associated responses [34], and phase I studies demonstrated tolerability in solid tumors [35, 36]. Our study extends these findings to MCC and provides a biological rationale for stratification: MCPyV-driven replication stress may help identify tumors more likely to respond to treatment. We also observed that WEE1 inhibition, although synergistic with gemcitabine in viability assays, induced weaker apoptotic responses than ATRi or CHK1i combinations. WEE1 functions downstream at the G2/M transition and may be less effective in cells arrested in the S-phase, where ATR-CHK1 is the dominant checkpoint. Under the conditions tested here, ATRi and CHK1i produced stronger pro-apoptotic responses than WEE1i when combined with gemcitabine, supporting our focus on the ATRi and CHK1i combinations in subsequent analyses.

### MCPyV status as a candidate stratification variable

The differential response between MCPyV^+^ and MCPyV^−^ models likely reflects differences in baseline replication-stress burden and in how DNA damage signaling couples to apoptosis. In MCPyV^+^ cells, ST-driven recruitment of Tip60-EP400 to L-Myc elevates MYC-dependent transcriptional programs and generates endogenous replication stress, creating a strong reliance on ATR-CHK1 signaling. This reliance may be reinforced by truncated LT-associated disruption of the Rb axis, which reduces G1/S checkpoint restraint and increases dependence on S-phase checkpoint control. In MCPyV^−^ cells, checkpoint perturbation induces replication-stress markers but fails to efficiently trigger apoptosis, and baseline drug sensitivity further shapes the response. Together, these findings support MCPyV status as a candidate stratification variable for ATR-CHK1-targeted combination therapy, although additional determinants, including intrinsic inhibitor sensitivity and the coupling of DDR signaling to apoptosis, are also likely to influence response.

### Role of Tip60-EP400 in replication stress

EP400 knockdown did not alter drug sensitivity when normalized for its proliferative effect, indicating that ATR-CHK1 inhibitor sensitivity persists even when Tip60-EP400 function is compromised. However, EP400 depletion attenuated CHK1i-induced checkpoint signaling, particularly p-CHK1 and p-KAP1, consistent with ST/L-Myc/Tip60-EP400-driven transcription contributing to endogenous replication stress. Under exogenous stress (CHK1i + HU), EP400-dependent differences in most markers were no longer apparent, likely because HU imposes a strong, transcription-independent replication stress that dominates over EP400-dependent signals. The selective persistence of attenuated p-KAP1 even under HU may reflect altered chromatin accessibility [38–40] and/or impaired Tip60-dependent ATM activation [50]. Overall, these data support a model in which Tip60-EP400 modulates the magnitude of replication stress signaling in MCPyV^+^ MCC, but does not independently determine sensitivity to ATR-CHK1 pathway inhibitors under the conditions tested. Additional viral and host factors, including ST functions independent of EP400 and truncated LT, likely also contribute.

### Clinical implications and future directions

MCPyV^+^ MCC patients who progress on immune checkpoint inhibitors may represent a rational population in which to evaluate replication stress directed combination therapy. In this context, the MATRiX trial is testing the ATR inhibitor tuvusertib with or without the PD-L1 inhibitor avelumab in advanced PD-L1-refractory MCC [51]. In parallel, Schnabel et al. showed that IMPDH inhibition induces replication stress in MCC and enhances the effects of ATR inhibition in vitro and in vivo [52]. Our findings complement these efforts by supporting gemcitabine as an alternative pharmacologic inducer of replication stress that can be paired with ATR or CHK1 pathway inhibition. Although the gemcitabine concentrations used in vitro are lower than reported peak plasma concentrations in patients, direct comparison is limited because clinical exposure is brief whereas cultured cells are exposed continuously [53, 54]. At the same time, both efficacy and toxicity are likely to depend on treatment schedule and drug context. All experiments in this study used simultaneous drug exposure. Prior work showed that delayed CHK1 inhibition after gemcitabine can enhance sensitization in some settings, whereas concurrent gemcitabine and ATR inhibition produced more durable growth suppression than gemcitabine pretreatment followed by ATR inhibition in another model [36, 55]. Normal cell effects also appear context dependent. The ATR inhibitor VE-822 did not enhance gemcitabine-induced killing in normal fibroblasts [56]. By contrast, gemcitabine combined with the CHK1 inhibitor GDC-0575 impaired proliferative potential in normal fibroblasts [57]. Another ATR inhibitor, ceralasertib (AZD6738), showed dose-dependent effects in fibroblasts, with pathway inhibition without apoptosis at low doses but apoptosis at higher concentrations [58]. These findings highlight the need to define both the optimal dosing schedule and the therapeutic window of gemcitabine combined with ATR-CHK1 pathway inhibition in MCC. Additional biomarkers beyond MCPyV status may further refine patient selection. Elevated ATR and CHK1 mRNA expression in MCPyV^+^ tumors, together with tumor replication stress markers, could help identify patients most likely to benefit [22].

### Limitations

Our experiments were performed in cell lines, and in vivo validation will be important. EP400 knockdown was achieved by short-term depletion, and prolonged loss may reveal different phenotypes. The relative contributions of ST, truncated LT, and Tip60-EP400 to replication stress remain to be fully dissected. Although platinum-based regimens with etoposide have historically been more commonly used in metastatic MCC [7], gemcitabine was selected here on mechanistic grounds as a replication stress inducer, and this study did not directly compare gemcitabine-based combinations with platinum-etoposide regimens. Germline DDR alterations have been identified in early-onset MCC patients [59], and whether such alterations influence sensitivity to ATR-CHK1-targeted combination therapy remains unknown. Direct genetic depletion of ATR or CHK1 in MCC cells would further strengthen evidence for on-target activity of the inhibitors used here. Earlier mechanistic time points were not assessed, which limits interpretation of the kinetics of target inhibition and apoptosis onset.

## Conclusion

Targeting ATR-CHK1 in combination with gemcitabine exploits replication-stress-associated vulnerabilities in MCPyV^+^ MCC and supports further preclinical and translational evaluation, particularly for patients who have progressed on immunotherapy.

## Data Availability

All data generated or analyzed during this study are included in this published article and its supplementary information files. Additional datasets or materials are available from the corresponding author upon reasonable request.

## References

[1] Lemos BD, Storer BE, Iyer JG, Phillips JL, Bichakjian CK, Fang LC, et al. Pathologic nodal evaluation improves prognostic accuracy in Merkel cell carcinoma: analysis of 5823 cases as the basis of the first consensus staging system. J Am Acad Dermatol. 2010;63(5):751–61. 10.1016/j.jaad.2010.02.056 PMC2956767 20646783

[2] Paulson KG, Iyer JG, Blom A, Warton EM, Sokil M, Yelistratova L, et al. Systemic immune suppression predicts diminished Merkel cell carcinoma-specific survival independent of stage. J Invest Dermatol. 2013;133(3):642–6. 10.1038/jid.2012.388 PMC3570636 23190897

[3] Zwijnenburg EM, Lubeek SFK, Werner JEM, Amir AL, Weijs WLJ, Takes RP, Pegge SAH, van Herpen CML, Adema GJ, Kaanders J. Merkel Cell Carcinoma: New Trends. Cancers (Basel). 2021;13(7):1614. 10.3390/cancers13071614. PMC8036880 33807446

[4] Becker JC, Stang A, DeCaprio JA, Cerroni L, Lebbe C, Veness M, et al. Merkel cell carcinoma. Nat Rev Dis Primers. 2017;3:17077. 10.1038/nrdp.2017.77 PMC6054450 29072302

[5] Heath M, Jaimes N, Lemos B, Mostaghimi A, Wang LC, Penas PF, et al. Clinical characteristics of Merkel cell carcinoma at diagnosis in 195 patients: the AEIOU features. J Am Acad Dermatol. 2008;58(3):375–81. 10.1016/j.jaad.2007.11.020 PMC2335370 18280333

[6] Gunaratne DA, Howle JR, Veness MJ. Definitive radiotherapy for Merkel cell carcinoma confers clinically meaningful in-field locoregional control: a review and analysis of the literature. J Am Acad Dermatol. 2017;77(1):142-148 e141. 10.1016/j.jaad.2017.02.015 28495499

[7] Iyer JG, Blom A, Doumani R, Lewis C, Tarabadkar ES, Anderson A, et al. Response rates and durability of chemotherapy among 62 patients with metastatic Merkel cell carcinoma. Cancer Med. 2016;5(9):2294–301. 10.1002/cam4.815 PMC5055152 27431483

[8] Nghiem P, Bhatia S, Lipson EJ, Sharfman WH, Kudchadkar RR, Brohl AS, et al. Durable tumor regression and overall survival in patients with advanced Merkel cell carcinoma receiving pembrolizumab as first-line therapy. J Clin Oncol. 2019;37(9):693–702. 10.1200/JCO.18.01896 PMC6424137 30726175

[9] de Moraes FCA, Kreuz M, de Lara ICA, Lobo AOM, Burbano RMR. Efficacy and safety of PD-1/PD-L1 inhibitors in patients with Merkel Cell Carcinoma: a systematic review and Meta-analysis. BMC Cancer. 2024;24(1):1357. 10.1186/s12885-024-13129-1 PMC11539798 39506680

[10] Starrett GJ, Thakuria M, Chen T, Marcelus C, Cheng J, Nomburg J, et al. Clinical and molecular characterization of virus-positive and virus-negative Merkel cell carcinoma. Genome Med. 2020;12(1):30. 10.1186/s13073-020-00727-4 PMC7081548 32188490

[11] Martel-Jantin C, Pedergnana V, Nicol JT, Leblond V, Tregouet DA, Tortevoye P, et al. Merkel cell polyomavirus infection occurs during early childhood and is transmitted between siblings. J Clin Virol. 2013;58(1):288–91. 10.1016/j.jcv.2013.06.004 23829968

[12] Tolstov YL, Knauer A, Chen JG, Kensler TW, Kingsley LA, Moore PS, et al. Asymptomatic primary Merkel cell polyomavirus infection among adults. Emerg Infect Dis. 2011;17(8):1371–80. 10.3201/eid1708.110079 PMC3381535 21801612

[13] Feng H, Shuda M, Chang Y, Moore PS. Clonal integration of a polyomavirus in human Merkel cell carcinoma. Science. 2008;319(5866):1096–100. 10.1126/science.1152586 PMC2740911 18202256

[14] Czech-Sioli M, Gunther T, Therre M, Spohn M, Indenbirken D, Theiss J, et al. High-resolution analysis of Merkel cell polyomavirus in Merkel cell carcinoma reveals distinct integration patterns and suggests NHEJ and MMBIR as underlying mechanisms. PLoS Pathog. 2020;16(8):e1008562. 10.1371/journal.ppat.1008562 PMC7470373 32833988

[15] Ma JE, Brewer JD. Merkel cell carcinoma in immunosuppressed patients. Cancers (Basel). 2014;6(3):1328–50. 10.3390/cancers6031328 PMC4190543 24978436

[16] Stakaityte G, Wood JJ, Knight LM, Abdul-Sada H, Adzahar NS, Nwogu N, et al. Merkel cell polyomavirus: molecular insights into the most recently discovered human tumour virus. Cancers (Basel). 2014;6(3):1267–97. 10.3390/cancers6031267 PMC4190541 24978434

[17] Goh G, Walradt T, Markarov V, Blom A, Riaz N, Doumani R, et al. Mutational landscape of MCPyV-positive and MCPyV-negative Merkel cell carcinomas with implications for immunotherapy. Oncotarget. 2016;7(3):3403–15. 10.18632/oncotarget.6494 PMC4823115 26655088

[18] Rotondo JC, Mazziotta C, Lanzillotti C, Tognon M, Martini F. Epigenetic Dysregulations in Merkel Cell Polyomavirus-Driven Merkel Cell Carcinoma. Int J Mol Sci. 2021;22(21):11464. 10.3390/ijms222111464 PMC8584046 34768895

[19] Cheng J, Park DE, Berrios C, White EA, Arora R, Yoon R, et al. Merkel cell polyomavirus recruits MYCL to the EP400 complex to promote oncogenesis. PLoS Pathog. 2017;13(10):e1006668. 10.1371/journal.ppat.1006668 PMC5640240 29028833

[20] Verhaegen ME, Mangelberger D, Harms PW, Vozheiko TD, Weick JW, Wilbert DM, et al. Merkel cell polyomavirus small T antigen is oncogenic in transgenic mice. J Invest Dermatol. 2015;135(5):1415–24. 10.1038/jid.2014.446 PMC4397111 25313532

[21] Cheng J, Rozenblatt-Rosen O, Paulson KG, Nghiem P, DeCaprio JA. Merkel cell polyomavirus large T antigen has growth-promoting and inhibitory activities. J Virol. 2013;87(11):6118–26. 10.1128/JVI.00385-13 PMC3648111 23514892

[22] Passerini S, Fracella M, Ferlosio A, Moens U, Scagnolari C, Antonelli G, et al. Investigation of mRNA expression levels of DNA damage response genes in Merkel Cell Polyomavirus-positive Merkel Cell Carcinoma: a pilot study. Discov Oncol. 2025;16(1):852. 10.1007/s12672-025-02651-8 PMC12095753 40399437

[23] Qiu Z, Oleinick NL, Zhang J. ATR/CHK1 inhibitors and cancer therapy. Radiother Oncol. 2018;126(3):450–64. 10.1016/j.radonc.2017.09.043 PMC5856582 29054375

[24] Sokka M, Koalick D, Hemmerich P, Syvaoja JE, Pospiech H. The ATR-Activation Domain of TopBP1 Is Required for the Suppression of Origin Firing during the S Phase. Int J Mol Sci. 2018;19(8):2376. 10.3390/ijms19082376 PMC6121618 30104465

[25] Buisson R, Boisvert JL, Benes CH, Zou L. Distinct but concerted roles of ATR, DNA-PK, and Chk1 in countering replication stress during S phase. Mol Cell. 2015;59(6):1011–24. 10.1016/j.molcel.2015.07.029 PMC4575890 26365377

[26] Zeman MK, Cimprich KA. Causes and consequences of replication stress. Nat Cell Biol. 2014;16(1):2–9. 10.1038/ncb2897 PMC4354890 24366029

[27] Carusillo A, Mussolino C. DNA Damage: From Threat to Treatment. Cells. 2020;9(7):1665. 10.3390/cells9071665 PMC7408370 32664329

[28] Buisson R, Niraj J, Rodrigue A, Ho CK, Kreuzer J, Foo TK, et al. Coupling of homologous recombination and the checkpoint by ATR. Mol Cell. 2017;65(2):336–46. 10.1016/j.molcel.2016.12.007 PMC5496772 28089683

[29] Wang M, Chen S, Ao D: Targeting DNA repair pathway in cancer: Mechanisms and clinical application. MedComm (2020). 2021;2(4):654–691. 10.1002/mco2.103 PMC8706759 34977872

[30] Bayat Mokhtari R, Homayouni TS, Baluch N, Morgatskaya E, Kumar S, Das B, et al. Combination therapy in combating cancer. Oncotarget. 2017;8(23):38022–43. 10.18632/oncotarget.16723 PMC5514969 28410237

[31] Tang Z, Pilie PG, Geng C, Manyam GC, Yang G, Park S, et al. ATR inhibition induces CDK1-SPOP signaling and enhances anti-PD-L1 cytotoxicity in prostate cancer. Clin Cancer Res. 2021;27(17):4898–909. 10.1158/1078-0432.CCR-21-1010 PMC8456453 34168048

[32] Ewald B, Sampath D, Plunkett W. H2AX phosphorylation marks gemcitabine-induced stalled replication forks and their collapse upon S-phase checkpoint abrogation. Mol Cancer Ther. 2007;6(4):1239–48. 10.1158/1535-7163.MCT-06-0633 17406032

[33] Hofer S, Frasch L, Brajkovic S, Putzker K, Lewis J, Schurmann H, et al. Gemcitabine and ATR inhibitors synergize to kill PDAC cells by blocking DNA damage response. Mol Syst Biol. 2025;21(3):231–53. 10.1038/s44320-025-00085-6 PMC11876601 39838187

[34] Konstantinopoulos PA, da Costa A, Gulhan D, Lee EK, Cheng SC, Hendrickson AEW, et al. A Replication stress biomarker is associated with response to gemcitabine versus combined gemcitabine and ATR inhibitor therapy in ovarian cancer. Nat Commun. 2021;12(1):5574. 10.1038/s41467-021-25904-w PMC8458434 34552099

[35] Daud AI, Ashworth MT, Strosberg J, Goldman JW, Mendelson D, Springett G, et al. Phase I dose-escalation trial of checkpoint kinase 1 inhibitor MK-8776 as monotherapy and in combination with gemcitabine in patients with advanced solid tumors. J Clin Oncol. 2015;33(9):1060–6. 10.1200/JCO.2014.57.5027 25605849

[36] Montano R, Thompson R, Chung I, Hou H, Khan N, Eastman A. Sensitization of human cancer cells to gemcitabine by the Chk1 inhibitor MK-8776: cell cycle perturbation and impact of administration schedule in vitro and in vivo. BMC Cancer. 2013;13:604. 10.1186/1471-2407-13-604 PMC3878047 24359526

[37] Paulson KG, Lemos BD, Feng B, Jaimes N, Penas PF, Bi X, et al. Array-CGH reveals recurrent genomic changes in Merkel cell carcinoma including amplification of L-Myc. J Invest Dermatol. 2009;129(6):1547–55. 10.1038/jid.2008.365 PMC2830552 19020549

[38] Chen PB, Hung JH, Hickman TL, Coles AH, Carey JF, Weng Z, et al. Hdac6 regulates Tip60-p400 function in stem cells. Elife. 2013;2:e01557. 10.7554/eLife.01557 PMC3843111 24302573

[39] Fazzio TG, Huff JT, Panning B. An RNAi screen of chromatin proteins identifies Tip60-p400 as a regulator of embryonic stem cell identity. Cell. 2008;134(1):162–74. 10.1016/j.cell.2008.05.031 PMC4308735 18614019

[40] Santopietro MV, Ferreri D, Prozzillo Y, Dimitri P, Messina G. The multitalented TIP60 chromatin remodeling complex: wearing many hats in epigenetic regulation, cell division and diseases. Epigenetics Chromatin. 2025;18(1):40. 10.1186/s13072-025-00603-8 PMC12220344 40604793

[41] Ravens S, Yu C, Ye T, Stierle M, Tora L. Tip60 complex binds to active Pol II promoters and a subset of enhancers and co-regulates the c-Myc network in mouse embryonic stem cells. Epigenetics Chromatin. 2015;8:45. 10.1186/s13072-015-0039-z PMC4636812 26550034

[42] Curti L, Campaner S. MYC-Induced Replicative Stress: A Double-Edged Sword for Cancer Development and Treatment. Int J Mol Sci. 2021;22(12):6168. 10.3390/ijms22126168 PMC8227504 34201047

[43] Dominguez-Sola D, Gautier J. MYC and the control of DNA replication. Cold Spring Harb Perspect Med. 2014;4(6):a014423. 10.1101/cshperspect.a014423 PMC4031955 24890833

[44] Sevigny JL, Proctor EV, Adams H, MacDonald ER, Thomas WK, Cheng J. Protein arginine methyltransferase 5 sustains Tip60-EP400 complex via SRSF1 in Merkel cell carcinoma. Life Sci Alliance. 2025;8(11):e202503316. 10.26508/lsa.202503316 PMC12373721 40846633

[45] Chou TC, Talalay P. Quantitative analysis of dose-effect relationships: the combined effects of multiple drugs or enzyme inhibitors. Adv Enzyme Regul. 1984;22:27–55. 10.1016/0065-2571(84)90007-4 6382953

[46] Matissek SJ, Han W, Karbalivand M, Sayed M, Reilly BM, Mallat S, et al. Epigenetic targeting of Waldenstrom macroglobulinemia cells with BET inhibitors synergizes with BCL2 or histone deacetylase inhibition. Epigenomics. 2021;13(2):129–44. 10.2217/epi-2020-0189 PMC8656319 33356554

[47] Menolfi D, Zha S. ATM, ATR and DNA-PKcs kinases-the lessons from the mouse models: inhibition not equal deletion. Cell Biosci. 2020;10:8. 10.1186/s13578-020-0376-x PMC6990542 32015826

[48] Liu S, Opiyo SO, Manthey K, Glanzer JG, Ashley AK, Amerin C, et al. Distinct roles for DNA-PK, ATM and ATR in RPA phosphorylation and checkpoint activation in response to replication stress. Nucleic Acids Res. 2012;40(21):10780–94. 10.1093/nar/gks849 PMC3510507 22977173

[49] Bachiri K, Kantar D, Laurent EMN, Gaboriaud P, Durand L, Drouin A, et al. DNA damage stress control is a truncated large T antigen and euchromatic histone lysine methyltransferase 2-dependent central feature of Merkel cell carcinoma. J Invest Dermatol. 2025;145(2):400-410 e404. 10.1016/j.jid.2024.04.034 38908781

[50] Sun Y, Jiang X, Chen S, Fernandes N, Price BD. A role for the Tip60 histone acetyltransferase in the acetylation and activation of ATM. Proc Natl Acad Sci U S A. 2005;102(37):13182–7. 10.1073/pnas.0504211102 PMC1197271 16141325

[51] Bhakuni R, Hall ET, Ansstas G, Bhatia S, Brohl AS, Burgess MA, et al. The MATRiX trial: a multicenter, randomized, phase II study of ATR inhibition (via tuvusertib) with or without avelumab in patients with advanced anti-PD-(L)1-refractory Merkel cell carcinoma. J Clin Oncol. 2025;43(16_suppl):TPS9598.

[52] Schnabel JL, Frost TC, Wang AC, Ananthapadmanabhan V, Gurram S, Soroko KM, et al. IMPDH inhibition induces DNA replication stress and ATR sensitivity in Merkel cell carcinoma. iScience. 2025;28(6):112567. 10.1016/j.isci.2025.112567 PMC12148403 40491496

[53] Ciccolini J, Serdjebi C, Peters GJ, Giovannetti E. Pharmacokinetics and pharmacogenetics of Gemcitabine as a mainstay in adult and pediatric oncology: an EORTC-PAMM perspective. Cancer Chemother Pharmacol. 2016;78(1):1–12. 10.1007/s00280-016-3003-0 PMC4921117 27007129

[54] Abbruzzese JL, Grunewald R, Weeks EA, Gravel D, Adams T, Nowak B, et al. A phase I clinical, plasma, and cellular pharmacology study of gemcitabine. J Clin Oncol. 1991;9(3):491–8. 10.1200/JCO.1991.9.3.491 1999720

[55] Wallez Y, Dunlop CR, Johnson TI, Koh SB, Fornari C, Yates JWT, et al. The ATR inhibitor AZD6738 synergizes with gemcitabine in vitro and in vivo to induce pancreatic ductal adenocarcinoma regression. Mol Cancer Ther. 2018;17(8):1670–82. 10.1158/1535-7163.MCT-18-0010 PMC6076438 29891488

[56] Fokas E, Prevo R, Pollard JR, Reaper PM, Charlton PA, Cornelissen B, et al. Targeting ATR in vivo using the novel inhibitor VE-822 results in selective sensitization of pancreatic tumors to radiation. Cell Death Dis. 2012;3(12):e441. 10.1038/cddis.2012.181 PMC3542617 23222511

[57] Oo ZY, Proctor M, Stevenson AJ, Nazareth D, Fernando M, Daignault SM, et al. Combined use of subclinical hydroxyurea and CHK1 inhibitor effectively controls melanoma and lung cancer progression, with reduced normal tissue toxicity compared to gemcitabine. Mol Oncol. 2019;13(7):1503–18. 10.1002/1878-0261.12497 PMC6599846 31044505

[58] Huang L, Ye Q, Lan C, Wang X, Zhu Y. AZD6738 inhibits fibrotic response of conjunctival fibroblasts by regulating checkpoint kinase 1/P53 and PI3K/AKT pathways. Front Pharmacol. 2022;13:990401. 10.3389/fphar.2022.990401 PMC9530343 36204234

[59] Mohsin N, Hunt D, Yan J, Jabbour AJ, Nghiem P, Choi J, et al. Genetic risk factors for early-onset Merkel cell carcinoma. JAMA Dermatol. 2024;160(2):172–8. 10.1001/jamadermatol.2023.5362 PMC10765310 38170500

